# Development of a Machine Learning Algorithm for the Surveillance of Autism Spectrum Disorder

**DOI:** 10.1371/journal.pone.0168224

**Published:** 2016-12-21

**Authors:** Matthew J. Maenner, Marshalyn Yeargin-Allsopp, Kim Van Naarden Braun, Deborah L. Christensen, Laura A. Schieve

**Affiliations:** 1 National Center on Birth Defects and Developmental Disabilities, Centers for Disease Control and Prevention; Atlanta, GA United States of America; 2 Epidemic Intelligence Service, Centers for Disease Control and Prevention; Atlanta, GA, United States of America; University of New South Wales, AUSTRALIA

## Abstract

The Autism and Developmental Disabilities Monitoring (ADDM) Network conducts population-based surveillance of autism spectrum disorder (ASD) among 8-year old children in multiple US sites. To classify ASD, trained clinicians review developmental evaluations collected from multiple health and education sources to determine whether the child meets the ASD surveillance case criteria. The number of evaluations collected has dramatically increased since the year 2000, challenging the resources and timeliness of the surveillance system. We developed and evaluated a machine learning approach to classify case status in ADDM using words and phrases contained in children’s developmental evaluations. We trained a random forest classifier using data from the 2008 Georgia ADDM site which included 1,162 children with 5,396 evaluations (601 children met ADDM ASD criteria using standard ADDM methods). The classifier used the words and phrases from the evaluations to predict ASD case status. We evaluated its performance on the 2010 Georgia ADDM surveillance data (1,450 children with 9,811 evaluations; 754 children met ADDM ASD criteria). We also estimated ASD prevalence using predictions from the classification algorithm. Overall, the machine learning approach predicted ASD case statuses that were 86.5% concordant with the clinician-determined case statuses (84.0% sensitivity, 89.4% predictive value positive). The area under the resulting receiver-operating characteristic curve was 0.932. Algorithm-derived ASD “prevalence” was 1.46% compared to the published (clinician-determined) estimate of 1.55%. Using only the text contained in developmental evaluations, a machine learning algorithm was able to discriminate between children that do and do not meet ASD surveillance criteria at one surveillance site.

## Introduction

Autism spectrum disorder (ASD) refers to a group of neurodevelopmental disorders characterized by impairments in social communication and repetitive behaviors and restricted interests. Like many other conditions described in the *Diagnostic and Statistical Manual of Mental Disorders (DSM)*, a diagnosis of ASD is based on the observation of behavioral features [1] and the specific cause of ASD is not known in many, if not most, cases. [2] A major challenge for ASD surveillance systems—and large studies of ASD in general—is reliable ascertainment of ASD.

Although rigorous ASD diagnostic instruments exist, clinicians use a variety of tools and approaches in everyday practice. [3] It is often infeasible for large-scale or population-based studies to classify ASD using the “gold-standard” practices used in clinical settings. Instead, many epidemiological studies rely—sometimes exclusively—upon existing “administrative” designations for ASD classification: International Classification of Diseases, 9^th^ Revision (ICD-9) billing codes, special education categories, or eligibility for disability benefits and services specific to autism (such as Medicaid). [4–9] In the United States, there is considerable variability in the utilization of these classifications and, because their primary intended purposes are to ensure appropriate service provision to individuals rather than to classify disabilities, these systems do not uniformly identify all individuals that meet ASD criteria in the population. [10–12]

To address the limitations of relying solely on existing codes or classifications for population-based tracking, the Centers for Disease Control and Prevention (CDC) developed a population-based ASD surveillance protocol that uses information from multiple health and education sources, and does not rely entirely upon existing ASD diagnoses or classifications. The Autism and Developmental Disabilities Monitoring (ADDM) Network uses a detailed process in which each site collects developmental evaluations from clinics and schools in their community. ADDM staff abstract verbatim descriptions from the evaluations, and experienced ADDM clinicians review children’s composite information to determine whether the descriptions of symptoms are consistent with ASD diagnostic criteria described in the *DSM*. [13] Approximately 20% of children meeting ADDM ASD criteria do not have a previously documented ASD diagnosis or classification. [14]

Given this labor-intensive review process, the timeliness and scope of the surveillance system are challenged by the continually-increasing volume of information that must be manually reviewed. While the overall reported prevalence of ASD has increased 120% between 2000 and 2010, there has been an even more dramatic increase in the annual number of evaluations that ADDM Network clinicians must review. For example, clinicians from the Georgia ADDM Network site reviewed 1,152 evaluations in 2000 and 9,811 in 2010—an increase of 750%.

To potentially improve the efficiency and timeliness of ASD surveillance, we developed and evaluated a machine learning-based algorithm that predicts whether a child will meet ASD surveillance criteria, using the words and phrases contained in a child’s evaluations.

## Methods

### ASD surveillance system and data

This study used data collected by the Metropolitan Atlanta Developmental Disabilities Surveillance Program, the Georgia site of the ADDM Network, from the 2008 and 2010 surveillance years. The study area covers five counties in metropolitan Atlanta. Following ADDM Network protocol, health and special education records for children aged 8 years and living in the study area during the surveillance year are requested from multiple clinics and schools in the community. Health records are requested if they are associated with certain ICD-9 billing codes, and special education records are requested from schools if the child is assigned to the autism special education eligibility category or to another category that might overlap with autism. These records are reviewed by trained record abstractors and, if ASD symptoms are present, all of a child’s developmental evaluations are copied into the surveillance database. Trained ADDM study clinicians review all abstracted evaluations and follow a protocol to code each evaluation for descriptions of *DSM-IV-TR* diagnostic criteria for pervasive developmental disorder-not otherwise specified or autistic disorder and also indicate whether the child had a previous ASD diagnosis. A clinician reviewer may classify a child’s record as an ASD case if there is sufficient description of the required number and pattern of behavioral features and the clinician decides ASD is an appropriate classification. For the purposes of achieving consensus and maintaining reliability, at least 10% of the records are reviewed by a second clinician. Additional reviews are also performed if a clinician reports a low degree of certainty about the ASD classification, and there is a defined process to reach a consensus. The clinician review process has high inter-rater reliability (90.7% agreement, kappa = 0.80), and a recent study demonstrated high sensitivity and specificity by having unaffiliated clinicians independently classify ADDM data. [14, 15] Additional details about the ADDM Network methods have been thoroughly described elsewhere. [13–19]

In 2008, the Georgia surveillance site abstracted 5396 evaluations for 1162 unique children; 601 children met the surveillance ASD case definition. In 2010, the surveillance dataset contained 1450 unique children with a total of 9811 evaluations; 754 children met the ASD case definition.

This study was submitted to the Centers for Disease Control and Prevention Institutional Review Board and was determined to be a non-research activity (public health practice) and was not required to undergo human subjects review.

### Processing text from evaluations

We used a “bag-of-words” approach, which captures the relative frequency of a word or phrase in a document, and disregards the order of the neighboring words or phrases. We extracted each child’s evaluations into a single body of text and removed punctuation symbols, numbers, converted letters to lower-case, and “stemmed” the words (removed word endings). We considered all words or phrases occurring in at least 3% (or 35 of 1162) of the children’s files. Instead of simply counting the frequency for each word or phrase, we used term-frequency—inverse-document frequency weighting (a common approach that considers both the frequency of a term in each child’s records and the proportion of children that have this term in their records). The resulting term-document matrix contained 1162 children (rows), and 13,135 1–3 word phrases (columns).

### Classification to predict ASD case status

We used random forests [20], an ensemble classification method, to accomplish two tasks. The first was to identify the subset of words and phrases that are most important for classifying ASD. The second task was to build an algorithm from the useful words and phrases to perform the actual classification. We developed (trained) the algorithm using only the 2008 surveillance data, and evaluated (tested) its predictive ability on the 2010 data.

We used random forests’ permutation-based variable importance scores to select the most important words. The scores represent the mean decrease in classification accuracy (over the entire forest) when the values for a word or phrase are replaced with random values. The change in accuracy is estimated by using the sub-sample of data that was not used to build a given tree (i.e., the “out-of-bag” sample). We selected the top 175 words and phrases to determine which to include in the final model (Fig A and Table A in S1 File).

Random forests generate many independently-grown decision trees, and the consensus vote of all the trees (‘the forest’) forms the final classification. (S1 Fig and S1 File) For a dichotomous classification, the default is to choose the outcome predicted by the majority of the trees. Because we have slightly fewer non-ASD than ASD cases, we adjusted the cut-off to reflect these proportions (561/1162 = 0.483 versus the default of 0.5). We also explored how alternate classification cut-offs affect performance. (Figs B and C in S1 File)

We used the algorithms developed with the 2008 data to classify 2010 data. We compared the algorithm to the final clinician-derived classification and calculated percent agreement, sensitivity, specificity, positive predictive value (PPV), and negative predictive value (NPV), Cohen’s kappa, and the area under the receiver-operating characteristic (ROC) curve. We also calculated ASD “prevalence” estimates based on the algorithm’s classifications. We performed all analyses with R 3.1.2 (R Foundation for Statistical Computing, Vienna, Austria), and in particular, the randomForest package. We generated a forest of 10,000 trees for the initial feature selection and forests of 3,000 trees for the reduced-feature set models, including the final model. We kept the default settings for all other parameters.

Finally, we compared the characteristics of children that were concordantly classified by the machine learning algorithm and by the clinicians to those that were discordant. We examined the proportion with a previously-documented ASD diagnosis (note that this is separate from the surveillance classification), number of evaluations collected for each child, sources of evaluations, demographic characteristics, and the proportion of children for whom the ADDM clinicians requested a secondary review (if they were uncertain about case status).

## Results

The final algorithm (using 90 terms) was trained on the 2008 Georgia ADDM data, and the terms are shown in Table A in S1 File. “Autism” was the single most important term, followed by phrases that include the word “autism” and references to symptoms such as eye contact or social interaction.

**Fig 1:**
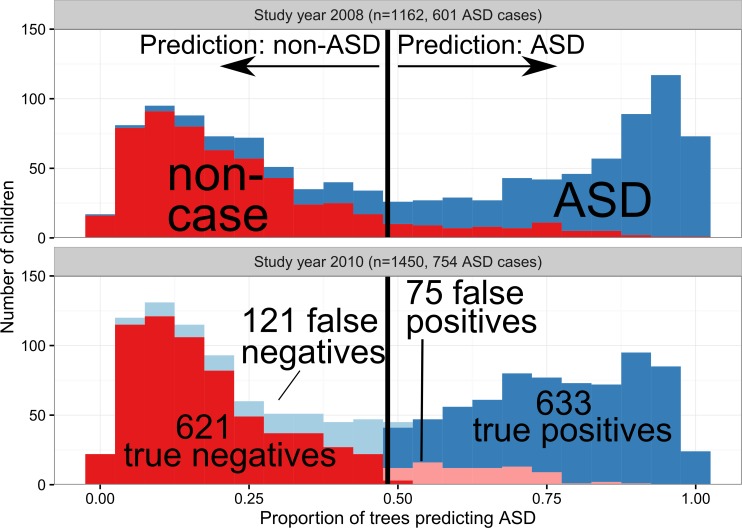
Histograms of prediction scores (x-axis) compared to clinician-assigned surveillance case definition (blue: autism spectrum disorder (ASD), red: non-ASD). Horizontal bar represents classification score threshold. Upper panel: classifications for 2008 (training) data. Bottom panel: 2010 (test) data, discordant classifications are highlighted in a lighter shade of blue or red.

We tested the model using 2010 Georgia ADDM surveillance data, which took one second to classify (after about three minutes to process the evaluations). The distributions of classification scores are shown in Fig 1, and Table 1 describes the summary performance measures. When applied to 2010 data, the algorithm agreed with the clinician-assigned case status for 86.5% of the 1,450 children (kappa = 0.73). Sensitivity, specificity, PPV and NPV for 2010 ranged from 83.7% to 89.4%. The area under the ROC curve was 0.932. We observed similar overall performance for the 2008 data.

**Table 1:** Comparison between clinician-assigned surveillance autism spectrum disorder (ASD) case status and predictions from random forest algorithm.

	Study Year	
	2008*	2010
Number of children abstracted	1162	1450
Number of ASD cases	601	754
*Comparison of algorithm to clinician classification*		
Simple Agreement (%)	86.3	86.5
Sensitivity (%)	84.5	84.0
Specificity (%)	88.2	89.2
Positive Predictive Value (%)	88.5	89.4
Negative Predictive Value (%)	84.2	83.7
Kappa	0.73	0.73
Area Under Receiver-Operating Characteristic Curve	0.932	0.932

Footnote: Autism spectrum disorder is abbreviated as ASD

*Note: the estimates for the 2008 data are calculated from the “out-of-bag” sample, reducing the potential for overfitting.

### Estimating ASD “prevalence” via algorithm

We compared previously-published ASD prevalence estimates [14] for the 2010 Georgia ADDM Network site to an estimate determined entirely by the algorithm, as shown in Table 2. Overall, the algorithm predicted that 708 of the 1450 children in the surveillance system would meet ASD case status, leading to an estimated prevalence of 14.6 per 1,000 children, approximately 6% lower than the previously-published estimate of 15.5 per 1,000. Similarly, most subgroup prevalence estimates for boys and racial/ethnic groups were all between 5–8% lower than-published estimates. For girls, the algorithm’s estimate was 11% lower than the previously-published estimate.

**Table 2:** Autism spectrum disorder (ASD) prevalence per 1,000 children (with 95% confidence interval) for 2010 Georgia Autism and Developmental Disabilities Monitoring Network site: comparison between published and algorithm-derived estimates.

Group	Published[14]		Algorithm-based		Algorithm: Published Prevalence Ratio
Overall	15.5	(14.5–16.7)	14.6	(13.6–15.7)	0.94
Boys	25.4	(23.5–27.5)	24.1	(22.3–26.1)	0.95
Girls	5.5	(4.6–6.5)	4.9	(4.1–5.9)	0.89
Non-Hispanic White	18.2	(16.2–20.4)	17.4	(15.5–19.5)	0.95
Non-Hispanic Black	14.0	(12.5–15.7)	13.0	(11.5–14.6)	0.93
Hispanic	10.7	(8.7–13.1)	10.1	(8.2–12.5)	0.94

### Characteristics of children with concordant and discordant classifications

The algorithm classification was discordant with the clinician-determined classification for 13.4% (195 of 1,450) of the children in the 2010 surveillance year (highlighted in lower panel of Fig 1). The characteristics of the concordant and discordant groups are described in Table 3. The “true-positive” (algorithm and clinician classified ASD, n = 633) differed from the “true-negative” (algorithm and clinician classified as non-ASD, n = 621) in several ways: “true-positives” had more evaluations (median of 7 versus 4), were more likely to have had a specific ASD or autism diagnosis or special education classification documented in their records (92.3% versus 4.7%), had a younger median age at first evaluation (40 versus 53 months), and were more likely to have evaluations from both school and non-school sources (65.9% versus 35.3%). The “false-positive” (algorithm: ASD, clinician: non-ASD, n = 75) group was similar to the “true-positive” group in terms of median number of evaluations (7), age at first evaluation (41 months), and proportion of children with both health and school sources (56.0%). In comparison, the “false-negative” (algorithm: non-ASD, clinician: ASD, n = 121) group was more similar to the “true-negative” group in terms of median number of evaluations (3), age at first evaluation (53months), and proportion of children with both health and school sources (38.8%). Previous ASD diagnoses or classifications were fairly common in both discordant groups (36.0% of false positives, and 44.6% of false-negatives).

**Table 3:** Characteristics of children, by algorithm-clinician concordance on autism spectrum disorder (ASD) case status.

	"True Positives"	"False Positives"	"False Negatives"	"True Negatives"
Clinician/Surveillance classification:	ASD	Non-ASD	ASD	Non-ASD
Algorithm prediction	ASD	ASD	Non-ASD	Non-ASD
Number of children (out of 1450)	633	75	121	621
Non-Hispanic White (%)	38.9 (35.1–42.7)	40.0 (30.0–51.3)	35.5 (27.6–44.4)	40.1 (36.3–44.0)
Male (%)	83.7 (80.1–86.4)	81.3 (71.1–88.5)	76.0 (67.7–82.8)	71.8 (68.2–75.2)
Known IQ < = 70 (%)	35.4 (31.8–39.2)	28.0 (19.1–39.0)	14.9 (9.6–22.3)	22.7 (19.6–26.2)
Previous diagnosis of autistic disorder (%)*	52.1 (48.2–56.0)	14.7 (8.4–24.4)	12.4 (7.7–19.4)	1.4 (0.7–2.7)
Previous ASD diagnosis other than autistic disorder (%)*	44.2 (40.4–48.1)	12.0 (6.4–21.3)	26.4 (19.4–34.9)	2.7 (1.7–4.3)
Previous ASD special education classification (%)*	65.4 (61.6–69.0)	12.0 (6.4–21.3)	15.7 (10.3–23.2)	0.8 (0.3–1.9)
Any autistic disorder/ASD diagnosis or ASD special education classification (%)	92.3 (89.9–94.1)	36.0 (26.1–47.3)	44.6 (36.1–53.5)	4.7 (3.3–6.6)
Number of evaluations (median and IQR)	7 (4–10)	7 (3–11)	3 (2–5)	4 (2–6)
Age in months at first evaluation (median and IQR)	40 (28–56)	41 (26–59)	53 (34–73)	53 (35–71)
Evaluations from school sources only (%)	22.4 (19.4–25.8)	30.7 (21.4–41.8)	36.4 (28.3–45.2)	43.0 (39.2–46.9)
Evaluations from health sources only (%)	11.7 (9.4–14.4)	13.3 (7.4–22.8)	24.8 (18.0–33.2)	21.6 (18.5–25.0)
Evaluation from both school and health sources (%)	65.9 (62.1–69.5)	56.0 (44.7–66.7)	38.8 (30.6–47.7)	35.3 (31.6–39.1)
ADDM reviewers requested a “secondary” review (%)**	11.4 (9.1–14.1)	64.0 (52.7–73.9)	44.6 (36.1–53.5)	31.7 (28.2–35.5)

Footnote: Autism spectrum disorder is abbreviated as ASD

*Note: Categories are not mutually exclusive; children often receive multiple diagnoses

**Note: Surveillance system clinician reviewers requested a second review from another clinician if they felt uncertain about a child’s ASD classification

ADDM clinicians were less likely to request secondary reviews (to resolve uncertainty) for children in the true-positives group (11.4%) than the true-negatives (31.7%). However, children in the two discordant groups had the highest proportions with secondary reviews (44.6% false negatives; 64.0% false positives). Figs D and E in S1 File illustrate how the algorithms scores compare to secondary reviews and clinician certainty scores.

## Discussion

These results demonstrate that a machine learning algorithm can discriminate between children that do and do not meet ASD surveillance criteria, among children with developmental concerns. Currently, the ADDM Network employs highly-trained clinicians to manually review each child’s developmental evaluations (often multiple evaluations per child), requiring an average of 45 to 60 minutes per child. Therefore, if the system must review an increasing number of records, it will require proportional increases in the resources needed to complete this task. In contrast, an automated approach requires relatively fixed resources for nearly any amount of information, and offers the potential to improve the efficiency and timeliness of the surveillance system.

Using only the words and phrases contained in a child’s records, the algorithm correctly predicted the clinician-assigned ASD case definition for 86.5% (kappa = 0.73) of the children captured by the surveillance system. This is slightly lower than the clinician inter-rater agreement observed for the overall 2010 ADDM Network (90.7%, kappa = 0.80). [14] Because the algorithm is trained on the clinician-assigned ratings, it is unlikely that agreement between the algorithm and a clinician would ever exceed inter-rater clinician agreement. On the other hand, the algorithm will have perfect intra-rater reliability, as it will always make the same classification for a given set of evaluations. An essential question is: what level of performance—if any—would be considered “acceptable” in order to trust the algorithm’s predictions? Of note, the algorithm-clinician agreement was similar to the inter-rater agreement reported by two other groups doing similar ASD classification on the basis of health records (one reported a kappa of 0.73 [21], and the other 88% agreement [22]).

The algorithm was more likely to misclassify children with certain characteristics. In particular, it was less sensitive to classifying ASD among children with fewer evaluations and those that were older when first evaluated. We also observed that the algorithm was more likely to misclassify children that underwent a secondary review by the ADDM clinicians (compared to those that did not undergo secondary review), suggesting these might be more difficult for the clinicians, as well. It might be possible to address some shortcomings by allowing the algorithm to consider the source or number of evaluations, or the age of the child at each evaluation. Alternately, the current model might serve as a useful “filter” to select the records that need manual review. As shown in Fig 1, the predictive values at the extreme ends of the range are quite high, with more misclassification in the middle. These scores could be used to identify records that need clinician reviews (e.g., a score of 0.50) versus those that are “safe bets” (e.g., scores over 0.80 or below 0.20). If, in the future, the surveillance system is able to electronically receive the contents of medical and educational evaluations, this type of “filter” could be immensely useful.

A previous study used an analogous approach using early intervention (birth to three years) records to predict which children would later be diagnosed with ASD. [23] The best-performing model from that study reported 91.4% precision (PPV) and 58.2% recall (sensitivity); the lower sensitivity is possibly due to a highly imbalanced ratio of ASD to non-ASD children. While this study had somewhat different goals from ours, the two studies suggest that text-based machine learning techniques may one day be useful in a variety of public health applications concerning ASD.

Other recent studies utilizing electronic health information have focused on using medical billing (ICD) codes to detect individuals with ASD. [24,25] These approaches are likely well-suited for case-control studies, where PPV might be more important than sensitivity, but will not detect individuals with ASD that do not have ICD codes. Because the algorithm we developed does not consider ICD-9 codes (as special education records do not assign them), the two approaches could be used to jointly classify ASD when both ICD-9 codes and evaluation text are available. In the future it may be possible to train classification algorithms on ADDM data and distribute them to help identify individuals with ASD from electronic records.

Although these results are promising, additional work is needed to evaluate the utility of this approach for ongoing ASD surveillance. For instance, performance characteristics—such as NPV or specificity—could be different in other populations. We trained the algorithm on a single year of data from one ADDM site and tested it on the following year’s data from the same site; we would need to evaluate whether similar performance could be achieved across ADDM sites or in other populations. We would also need to monitor performance so that it does not drift or degrade over time. In particular, the relatively recent changes to the ASD diagnosis in the *DSM-5* could affect the terms used to describe ASD symptoms. Likewise, the surveillance case definition for the ADDM Network may change to reflect the *DSM-5* criteria. For these reasons—and others—it is likely that any long-running system would require some level of continued manual review to assess the performance and quality of the system. Nevertheless, even a partially automated approach—in which a clinician might confirm or augment the algorithm’s predictions—could result in a substantial reduction in required resources.

The ADDM clinicians currently code a variety of behavioral symptoms and produce much more information than a dichotomous case classification; it remains to be seen whether these methods could reliably classify specific symptoms in addition to the overall ASD classification. We plan to pursue much more granular and classification algorithms for specific symptoms or for different populations were our current performance was weakest (such as girls, children only seen after age 6, or children without an intellectual disability). It would also be useful to estimate how well the algorithm (and the ADDM methods in general) compare to other ASD classifications, such as in-person assessments. Quantifying this textual information in a reproducible way will provide novel opportunities to better understand how children are evaluated for ASD in typical community settings.

This study is based on a large, population-based surveillance system that has routinely performed ASD surveillance in metropolitan Atlanta for more than a decade. The ASD surveillance case definition uses a well-established protocol for ascertaining ASD from record review, including extensive documentation, training materials, and inter-rater reliability for this procedure. As a by-product of conducting surveillance, the ADDM Network generates information that is useful for training text-based ASD classification algorithms. With relatively small modifications, it could efficiently produce a large volume of very specific examples that could be used to identify particular symptoms or behaviors. Ultimately, the approach piloted in this study could be trained on a much larger sample representing a diversity of community providers and behavioral evaluations.

## Conclusion

Public health surveillance systems are constantly challenged to become faster, better, or to provide the same information for lower cost. [26] We observed that an automated approach could predict—with high agreement—whether a child would meet ASD surveillance criteria. While there are many logistical issues to consider, these results hint at the potential for using machine learning approaches to identify ASD from unstructured text data.

## Supporting Information

S1 Fig Application of Random Forests to Autism Surveillance Data. (PDF)

S1 File Random Forests: identifying important words and phrases and classification cutoffs. **Fig A: Plot of random forest (RF) importance scores versus rank of importance scores for 13,135 words and phrases in 2008 training dataset.** Vertical line is drawn at the 175th-most important term. **Table A: Most important terms in final random forest model. Fig B: Accuracy of model when different classification cut-points are selected. Fig C: Sensitivity (red) and positive predictive value (PPV) (blue) at different cut-off thresholds.** Vertical line shows the classification threshold used in the final model. **Fig D: Algorithm classification scores and clinician certainty scores for autism spectrum disorder (ASD) surveillance. Fig E: Algorithm classification scores and Autism and Developmental Disabilities Monitoring (ADDM) Network clinician requests for a secondary review.**
(DOCX)
