# Supplementary material for: Development of a Machine Learning Algorithm for the Surveillance of Autism Spectrum Disorder
Source: PLoS One. 2016 Dec 21;11(12):e0168224. doi: 10.1371/journal.pone.0168224 (PMC5176307; doi:10.1371/journal.pone.0168224)
Supplement: S1 Fig — (PDF) [file pone.0168224.s001.pdf]

Application of random forest algorithm to autism spectrum disorder (ASD) surveillance data

1. Random forests: training one tree

Sample N observations
with replacement
(leaves out ~37%, or 1/e)

2008 Training Data
N = 1,162 Children
K = 13,135 phrases

2. Training one tree (cont'd)

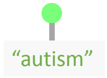

2. Random subset of sqrt(K) words/phrases; choose term that best separates outcomes

3. Training one tree (cont'd)

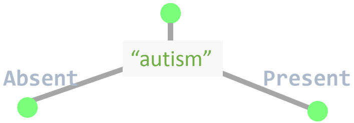

3. **Split sample** using the values of the selected term

4. Training one tree (cont'd)

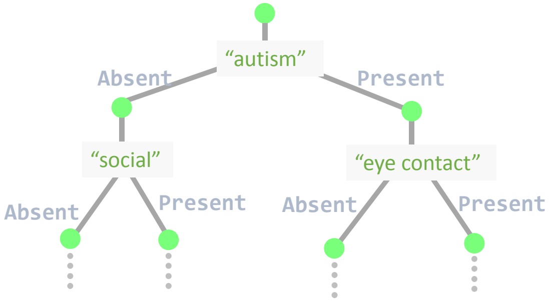

Repeat selection and splitting until tree is fully grown.

5. Classification

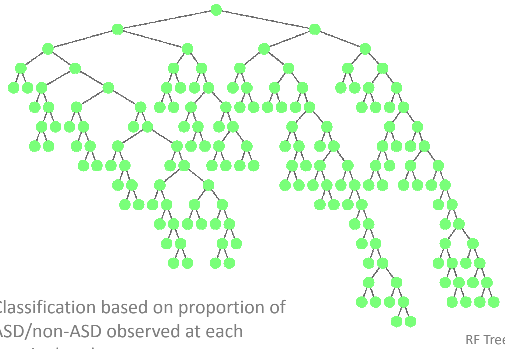

Classification based on proportion of ASD/non-ASD observed at each terminal node

RF Tree
1 of 10,000

6. Voting on ASD case status

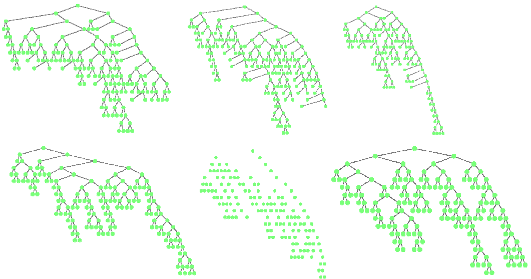

Each tree predicts every child's ASD case status.

Child's classification score $= \frac{1}{nTree}\sum_{i=1}^{nTree}(Prediction_i)$
