# Supplementary material for: Development of a Machine Learning Algorithm for the Surveillance of Autism Spectrum Disorder
Source: PLoS One. 2016 Dec 21;11(12):e0168224. doi: 10.1371/journal.pone.0168224 (PMC5176307; doi:10.1371/journal.pone.0168224)
Supplement: S1 File — Fig A: Plot of random forest (RF) importance scores versus rank of importance scores for 13,135 words and phrases in 2008 training dataset. Vertical line is drawn at the 175th-most important term. Table A: Most important terms in final random forest model. Fig B: Accuracy of model when different classification cut-points are selected. Fig C: Sensitivity (red) and positive predictive value (PPV) (blue) at different cut-off thresholds. Vertical line shows the classification threshold used in the final model. Fig D: Algorithm classification scores and clinician certainty scores for autism spectrum disorder (ASD) surveillance. Fig E: Algorithm classification scores and Autism and Developmental Disabilities Monitoring (ADDM) Network clinician requests for a secondary review. (DOCX) [file pone.0168224.s002.docx]

**Appendix 2: Random Forests: identifying important words and phrases and classification cutoffs**

**Random Forests (see illustration in Appendix 1):**

For each tree, a sample is drawn—with replacement--whose size is equal to the number of observations in the data set. Therefore, different trees are built with different sub-samples of the data. At each node in the tree, a small sample of words or phrases (equal to the square root of the total number words/phrases) are randomly selected, and the word/phrase that best separates individuals with and without autism spectrum disorder (ASD) is used to split the data into two groups. This process repeats until the tree is fully grown. Each tree in the forest predicts the ASD case status for every child, and the final prediction is based on the proportion of trees that classify the child as having ASD.

A brief explanation of the “out of bag sample”. Each tree is grown with a random sample of data (drawn with replacement) equal to the total number of observations in the data set. Therefore, on average, 1/e or ~37% of the samples are *not* used to train a given tree. These samples are called “out of bag” samples, and we estimated the classification error using the out of bag samples for the 2008 data set. (i.e., each observation in the 2008 dataset would be, on average, part of the out-of-bag sample for 37% of the trees—all samples in the dataset are part of the out-of-bag sample at some point).

Because we observed nearly the same result in the 2010 dataset (which was not used to grow the tree), we do not believe that the algorithm was overfitting to the 2008 data.

**First Random Forest algorithm: calculating importance scores for ALL words and phrases:**

**Figure A: Plot of random forest (RF) importance scores versus rank of importance scores for 13,135 words and phrases in 2008 training dataset.**


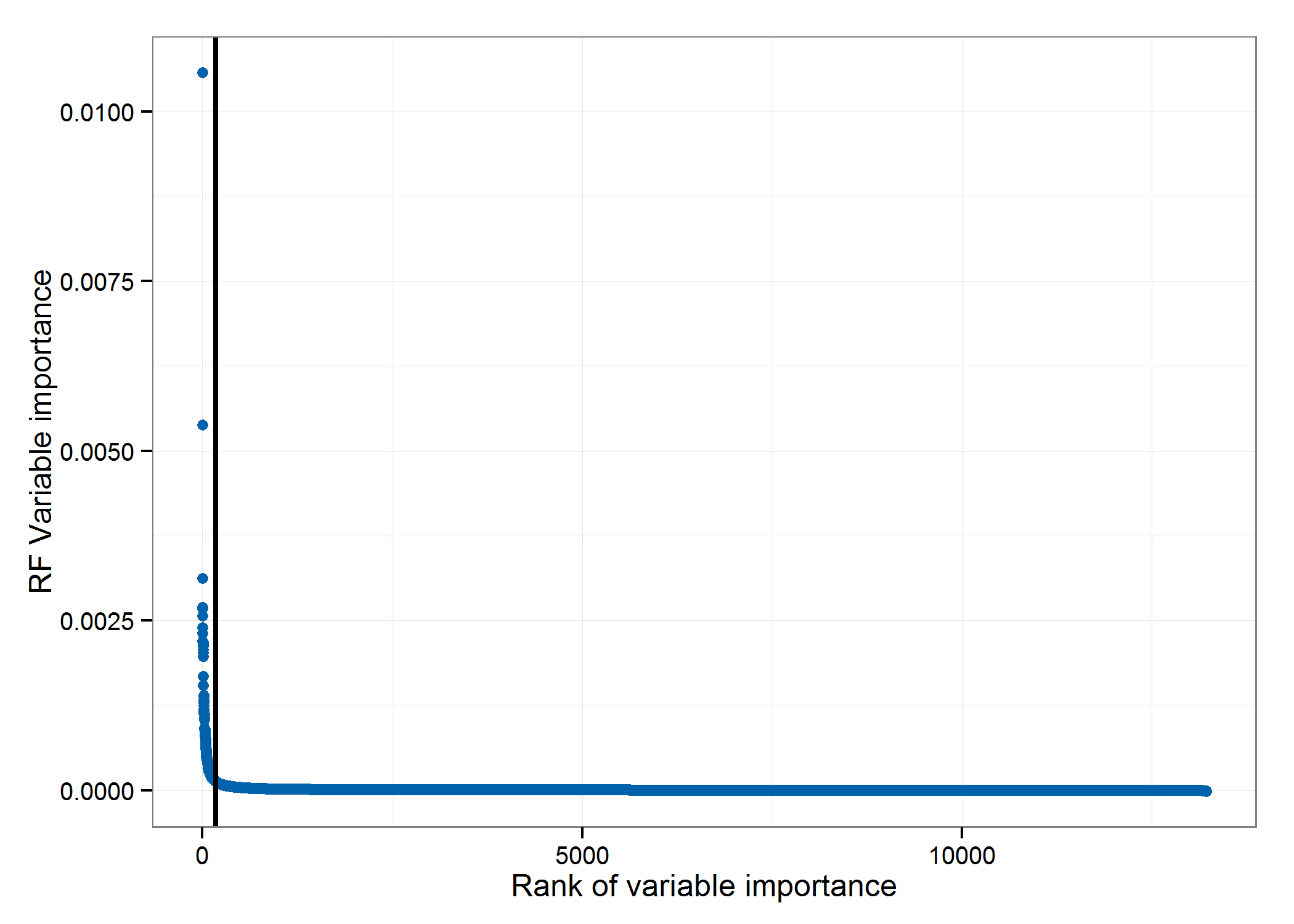


Vertical line is drawn at the 175^th^-most important term.

Footnote: RF is an abbreviation for random forest.

**Comment:** The figure shows a sharp decrease in importance starting with the first few words, and most of the words have importance scores that are nearly indistinguishable from zero—that is, they have no effect on classification accuracy. Removing “non-informative” terms from the classification algorithm is advantageous for two reasons: first, random forests often perform slightly better after excluding “nuisance” features which gives the trees a higher probability of choosing informative features. Second, it reduces the dependency on having specific words appear in future evaluations (such as 2010 data or – in the future—data from other surveillance sites).

The top 175 terms were considered by the varSelRF R package for selection into the final model. It produces a series of random forests using the 2008 study year data, eliminating the words with importance scores in the bottom 20% in a step-wise fashion. (From 175 to 140 to 112 to 90 to 72… etc) After each iteration it measures the (out-of-bag) error for the model and calculates the importance scores to drop the least important variables. We had the process stop when the model error increased in the next iteration (by more than a small margin). Although the out-of-bag error was relatively flat across the range, it was lowest at 90 terms and then started to increase after more terms were removed, so we used the selected 90 words and phrases in our final model (on next page).

These words include 1, 2, or 3-word phrases that were stemmed (removes suffixes) and set to lowercase. Some words appear multiple times on the list—alone and part of phrases. For instance, the list includes the terms “eye.contact”, “eye”, and “contact”. It is possible that this “double-counting” of words leads to sub-optimal performance, although we did observe that using 2-3 word phrases was better than only using single words. We plan to pursue more sophisticated phrase-detection methods in the future.

**Table A. Most important terms in final random forest model**

| \| **Term** \| **MeanDecreaseAccuracy** \| \| --- \| --- \| \| autism \| 0.053333 \| \| autism.elig \| 0.019117 \| \| social.interact \| 0.01034 \| \| doe.meet.autism \| 0.009253 \| \| eye.contact \| 0.008982 \| \| of.autism \| 0.008823 \| \| eye \| 0.007012 \| \| autist \| 0.006326 \| \| spectrum \| 0.006177 \| \| meet.autism \| 0.005953 \| \| interact \| 0.004934 \| \| autism.elig.report \| 0.004589 \| \| autism.spectrum \| 0.004401 \| \| pdd \| 0.004329 \| \| meet.autism.elig \| 0.004221 \| \| social.interact.and \| 0.004152 \| \| contact \| 0.003918 \| \| repertoir.of \| 0.003876 \| \| spin \| 0.003652 \| \| with.autism \| 0.003223 \| \| sensori \| 0.002968 \| \| echolalia \| 0.002726 \| \| space \| 0.002665 \| \| interest \| 0.002549 \| \| interact.and \| 0.00243 \| \| flap \| 0.0023 \| \| student.doe.meet \| 0.002265 \| \| autism.elig.criteria \| 0.002233 \| \| month \| 0.002231 \| \| object \| 0.002172 \| \| repetit \| 0.002167 \| \| doe.meet \| 0.002157 \| \| use \| 0.002104 \| \| communic \| 0.002096 \| \| elig \| 0.002075 \| \| interact.and.particip \| 0.001989 \| \| autism.and \| 0.001962 \| \| delay \| 0.001913 \| \| autist.disord \| 0.00191 \| \| play \| 0.001874 \| \| social \| 0.001874 \| \| he.did.not \| 0.001809 \| \| hand \| 0.001787 \| \| pervas.development \| 0.001752 \| \| chang \| 0.001733 \| \| tend \| 0.001683 \| \| transit \| 0.001604 \| \| spectrum.disord \| 0.001586 \| \| line.up \| 0.001568 \| \| smell \| 0.001554 \| \| repertoir \| 0.00153 \| \| problem \| 0.001461 \| \| numberof.month \| 0.001461 \| \| nois \| 0.001453 \| \| pattern.of.behavior \| 0.001422 \| \| development.disord \| 0.00139 \| \| numberof.numberof.month \| 0.00138 \| \| reciproc \| 0.001379 \| \| elig.criteria \| 0.001363 \| \| languag \| 0.001355 \| \| activ \| 0.00134 \| \| in.his \| 0.001324 \| \| student.doe \| 0.001313 \| \| sensori.process \| 0.001286 \| \| asd \| 0.001286 \| \| was.refer \| 0.001285 \| \| he.tend.to \| 0.001267 \| \| doe \| 0.001267 \| \| the.student.doe \| 0.001238 \| \| he.did \| 0.001181 \| \| word \| 0.00116 \| \| autism.spectrum.disord \| 0.001149 \| \| of.activ \| 0.001133 \| \| stereotyp \| 0.001109 \| \| criteria \| 0.001107 \| \| learn \| 0.001073 \| \| car \| 0.00105 \| \| initi \| 0.001031 \| \| such.as \| 0.000935 \| \| and.particip \| 0.000919 \| \| recept \| 0.000905 \| \| of.pdd \| 0.000904 \| \| routin \| 0.000888 \| \| particip \| 0.000884 \| \| his.hand \| 0.000867 \| \| doe.not.like \| 0.000857 \| \| did.not \| 0.000813 \| \| prefer \| 0.000787 \| \| watch \| 0.000615 \| \| characterist \| 0.000511 \| |  |
| --- | --- | --- | --- | --- | --- | --- | --- | --- | --- | --- | --- | --- | --- | --- | --- | --- | --- | --- | --- | --- | --- | --- | --- | --- | --- | --- | --- | --- | --- | --- | --- | --- | --- | --- | --- | --- | --- | --- | --- | --- | --- | --- | --- | --- | --- | --- | --- | --- | --- | --- | --- | --- | --- | --- | --- | --- | --- | --- | --- | --- | --- | --- | --- | --- | --- | --- | --- | --- | --- | --- | --- | --- | --- | --- | --- | --- | --- | --- | --- | --- | --- | --- | --- | --- | --- | --- | --- | --- | --- | --- | --- | --- | --- | --- | --- | --- | --- | --- | --- | --- | --- | --- | --- | --- | --- | --- | --- | --- | --- | --- | --- | --- | --- | --- | --- | --- | --- | --- | --- | --- | --- | --- | --- | --- | --- | --- | --- | --- | --- | --- | --- | --- | --- | --- | --- | --- | --- | --- | --- | --- | --- | --- | --- | --- | --- | --- | --- | --- | --- | --- | --- | --- | --- | --- | --- | --- | --- | --- | --- | --- | --- | --- | --- | --- | --- | --- | --- | --- | --- | --- | --- | --- | --- | --- | --- | --- | --- | --- | --- | --- | --- | --- | --- |
|  |  |

**Selecting classification cut-off thresholds**In the paper, we chose a classification cut-off slightly different from 0.50 because there is an unequal number of ASD cases and non-cases in the training data. We set the threshold at 0.483, to reflect the proportion of non-ASD cases. Because this is an arbitrary decision, we investigated whether a different cutoff would give different performance.

We examined agreement (in the 2008 training data) at every cutoff (in increments of 0.001), plotted in the figure below (represented by the arc-shaped line). The dark vertical line is our a priori cutoff at .483, and the red dashed line is the “optimal” cutoff (based on the 2008 training data) at 0.409. These overall agreements are all quite similar to each other, so we used the initial, predetermined, cutoff value of 0.483 in the paper.

**Figure B: Accuracy of model when different classification cut-points are selected.**
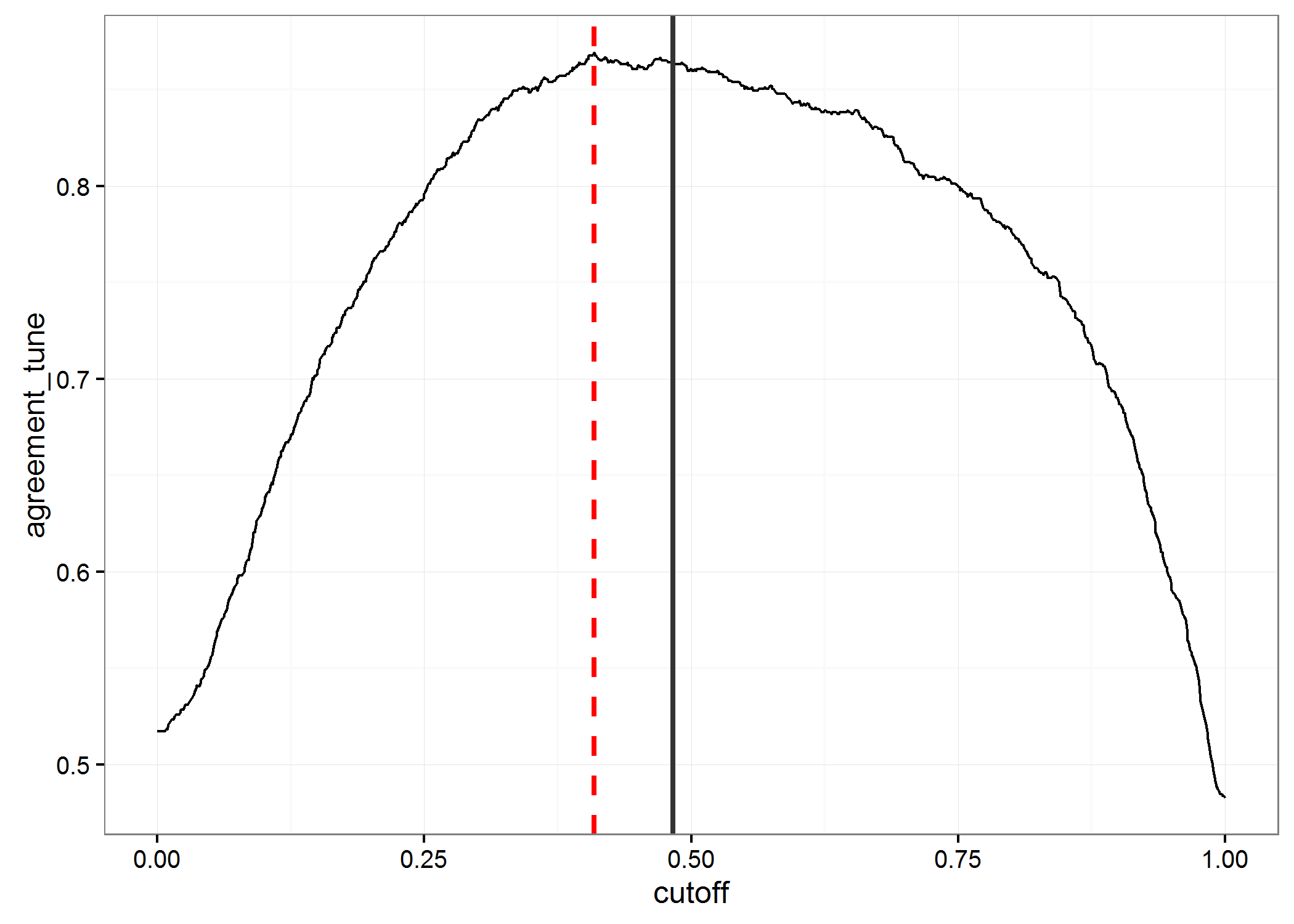


| Cutoff | Overall percent agreement |
| --- | --- |
| 0.483 (proportion of non-cases) | 86.4 |
| 0.409 (“optimal” based on data) | 86.9 |
| 0.500 (default RF for binary classification) | 86.3 |

Because these values are so similar, we left the cutoff at our original value. However, this approach could be used to “tune” future statistical learning classifiers.

**Figure C: Sensitivity (red) and positive predictive value (PPV) (blue) at different cut-off thresholds.**


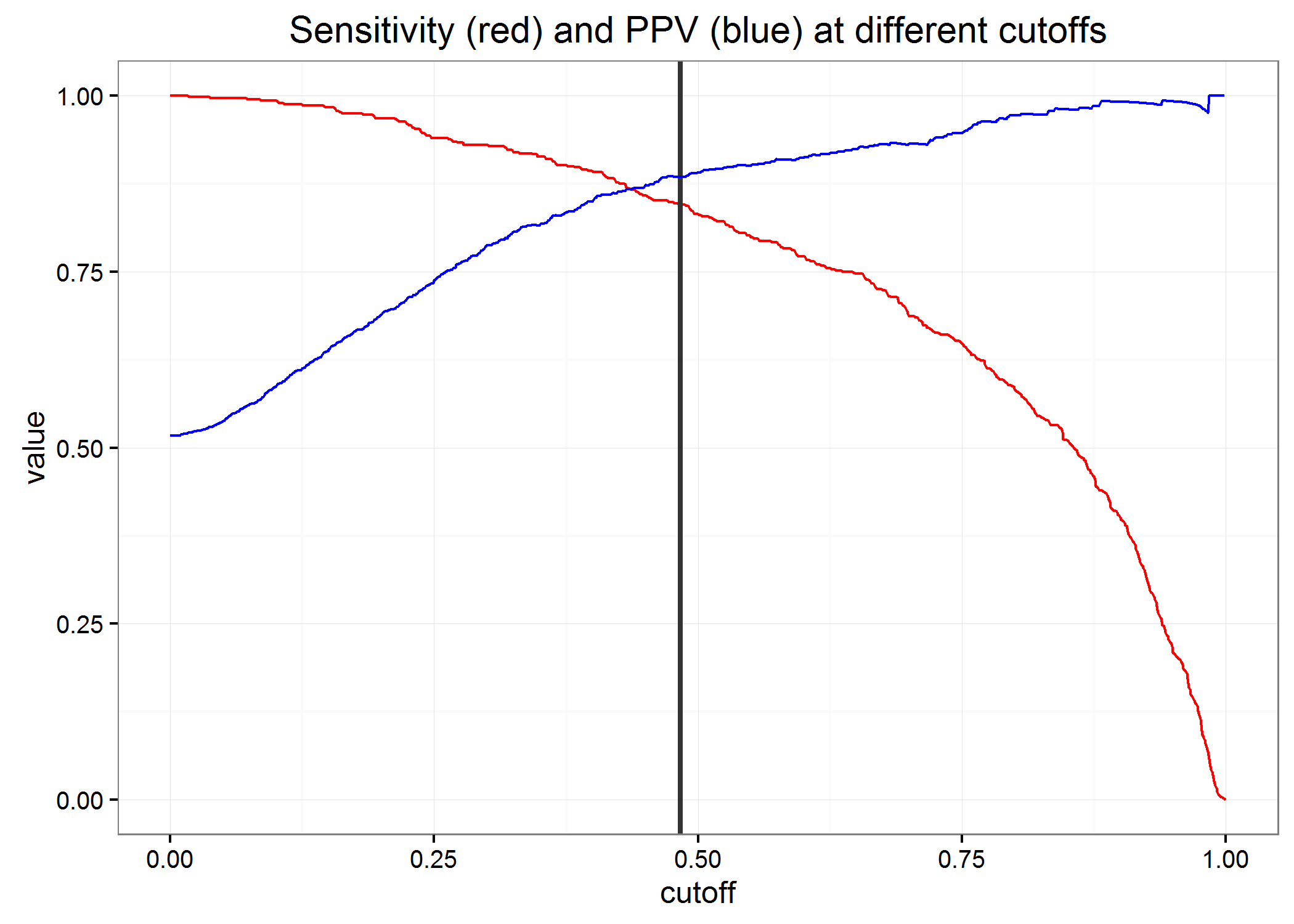


Vertical line shows the classification threshold used in the final model.

**Algorithm classification scores and clinician uncertainty**

**Figure D: Algorithm classification scores and clinician certainty scores for autism spectrum disorder (ASD) surveillance**

**
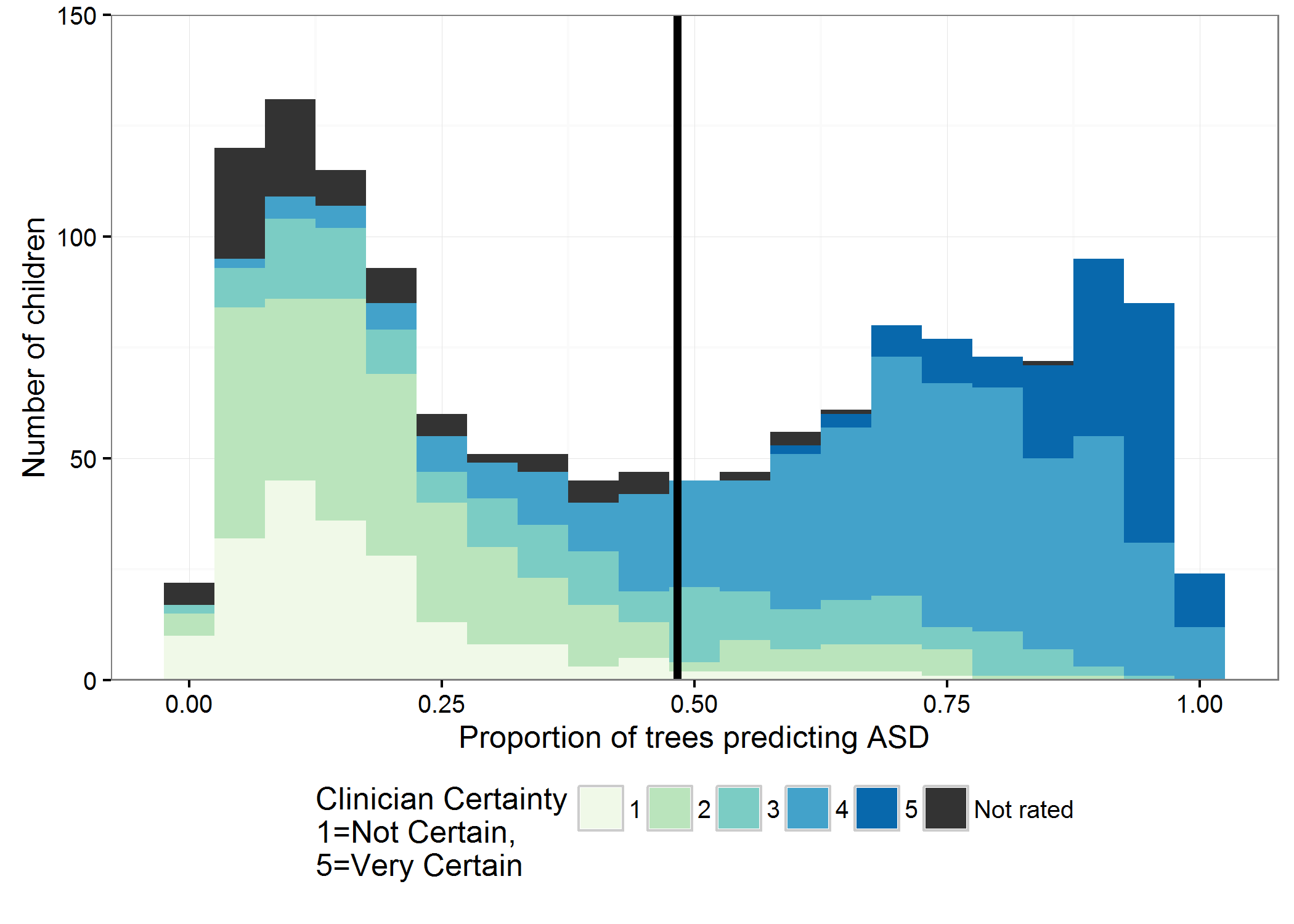
**

**Comment:** Clinicians rated their certainty that a child was an ASD case on a 1-5 scale (with 5 being most certain that the child should be classified as having ASD). The unrated children (shown in gray) are those that had the least amount of ASD symptoms and the clinicians ruled out the possibility that a child could be classified as having ASD.

**Figure E: Algorithm classification scores and Autism and Developmental Disabilities Monitoring (ADDM) Network clinician requests for a secondary review.**


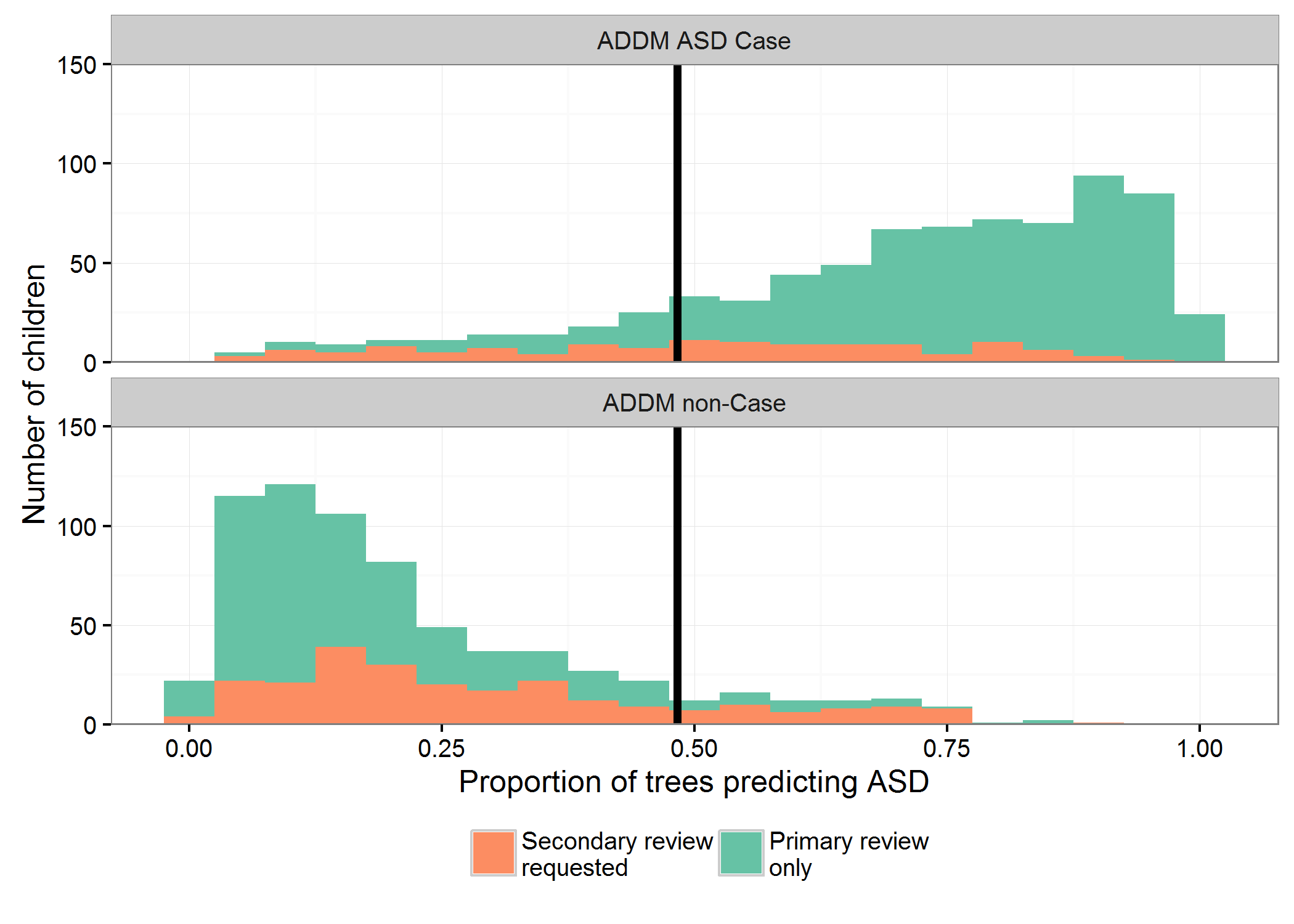


**Comment:** When clinicians felt uncertain about a child’s classification, they could request a secondary review. This figure shows the distribution of children with secondary versus primary-only reviews over the range of algorithm classifications scores. The figure is stratified into two groups: children that were classified as having ASD by ADDM clinicians (Upper panel), and children that did not meet ADDM ASD case criteria (lower panel). As summarized in Table 3 in the manuscript, the children with the highest algorithm scores had relatively few secondary reviews from the clinicians. In contrast, 65% (49 / 75) of children classified as “false positives” (lower panel, to the right of the vertical line) underwent a secondary review, suggesting that many of these were also challenging for the clinicians to classify. Of these 49 children undergoing secondary review, 48 were determined to have symptoms consistent with ASD by at least one clinician, but were ultimately ruled out because the clinicians felt the symptoms could be better explained by another disorder.
